# Reduced Balance Restoration Capacities Following Unilateral Vestibular Insult in Elderly Mice

**DOI:** 10.3389/fneur.2018.00462

**Published:** 2018-06-25

**Authors:** Raphaelle Cassel, Sylvette Wiener-Vacher, A. El Ahmadi, Brahim Tighilet, Christian Chabbert

**Affiliations:** ^1^Laboratoire de Neurosciences Sensorielles et Cognitives - Equipe physiopathologie et Thérapie des Désordres Vestibulaire, Centre National de la Recherche Scientifique, Aix Marseille Université, UMR 7260, Marseille, France; ^2^Laboratoire d'Exploration Fonctionnel de l'Équilibre chez l'Enfant, APHP, Université Paris VII, Paris, France

**Keywords:** acute vestibular syndrome, equilibrium impairment, vestibular deafferentation, behavioral evaluation, mouse model

## Abstract

Acute vestibular syndrome (AVS) is characterized by severe posturo-locomotor and vestibulo-oculomotor impairment and accompanies several types of peripheral vestibulopathies (PVP). We know very little about its etiology, how its various symptoms are expressed and how it evolves with age. Robust repair capabilities of primary vestibular synapses have recently been shown to restore behavioral functionality. In this study, we used a mouse model of an excitotoxically induced unilateral vestibular lesion to compare the ability to restore balance and posture between old and young adult mice. We compared the temporal evolution of the evoked vestibular syndrome using a battery of behavioral tests to follow the evolution of postural-locomotor alterations and equilibrium. For the first time, we show that young adult (3 months) and elderly (22 months) mice are together able to restore normal postural-locomotor function following transient unilateral excitotoxic vestibular insult, though with different time courses. This animal study paves way for future, more detailed studies of how the early postural and locomotor disturbances following a unilateral insult are compensated for by various plasticity mechanisms, and in particular how age influences these mechanisms.

## Introduction

Acute vestibular syndrome (AVS) expressed in peripheral vestibulopathy (PVP) is characterized by sustained rotatory vertigo; postural imbalance with Romberg's sign (i.e., falls toward the affected ear with eyes closed) at rest and during movement; ipsilateral lateropulsion and tilted body posture; spontaneous nystagmus beating toward the unaffected ear; and ipsilateral ocular tilt reaction and oscillopsia, accompanied by nausea (1, 2). This syndrome is frequently expressed in vestibular neuritis and labyrinthitis (3, 4). AVS can be highly disabling and may last several days, while chronic disabilities and clinical deficits often persist for several years (5, 6). Caloric testing (irrigation of the ear with warm or cold water) invariably shows ipsilateral hyporeflexia or areflexia (4). Following its acute phase, the vestibular syndrome subsides in a process of behavioral recovery known as vestibular compensation. The static symptoms include ocular motor (spontaneous nystagmus) and postural (head tilt, support surface enhancement) deficits that are compensated within a few days or weeks, depending on the species, while the dynamic symptoms (vestibulo-ocular reflex, dynamic equilibrium function) are compensated much less completely or over a longer period (7–9). The static deficits result from the spontaneous resting activity imbalance between the bilateral vestibular nucleus complexes (VNCs) and its compensation, that is, restoration of a balanced electrical activity between the VNCs. This was demonstrated electrophysiologically in different animal models of unilateral vestibular injury (10–12). By contrast, compensation of dynamic symptoms does not appear to be dependent on a return to electrophysiological homeostasis between the two VNCS but on a multitude of plasticity mechanisms that develop both in the two VNCS but also in other structures of the central nervous system (7, 13–20).

No etiology is currently accepted to explain the generation of PVP. Whether viral or ischemic causes have been proposed to explain the particular symptomology of PVP, evidences are insufficient to point out a single or even preferential cause to these pathologies. Neither the biological analyses carried out in VN patients, nor post mortem histological analyses allowed to determine a unique and irrefutable pathogenic cause. Classical clinical evaluation tests (caloric test, VEMP, VHIT) also failed to accurately and definitely identify the area of the inner ear damaged in PVP. Similarly, MRI approaches, looking for a hyper-signal attributable to inflammatory foci along the vestibular nerve, validated this possibility only in 3% (over 70 patients) of VN patients (21–23). In 97% of cases the damaged area could not be determined. In cases of sudden sensory neural hearing loss (SSNHL), initially also attributed to inner ear inflammatory damages, it is now considered that the vast majority of damage is of intracochlear origin. This has been validated by the detection of hyper-signal in MRI (24). Contacts between auditory sensory cells and auditory nerve neurons are considered to be the most fragile area of the inner ear (25). Selective damage to the primary synapses could support all of the symptoms experienced in SSNHL. It can be assumed that similar situation may occur in labyrinth.

In order to directly address this question, we previously developed a rodent model of AVS by inducing unilateral excitotoxic damage to the inner ear sensory organs by means of trans-tympanic administration of the glutamate receptor agonist kainate [TTK administration model; (26)]. This model allowed us to identify the time course of the different postural-locomotor symptoms that compose AVS. It also highlighted the ability of rodents to quickly restore their balance and posture, based on a robust propensity of peripheral vestibular synapses to spontaneously repair following kainate-induced deafferentation (27). A recent adaptation of the mouse TTK model and the development of more precise monitoring of both the specific signs of vestibular function and the general behavioral alterations provided the opportunity to better follow the temporal evolution of several specific symptoms of AVS, while better appreciating the different phases that compose this syndrome (28). It also brought insights into our understanding on how AVS develops and how it affects global behavior.

Several recent epidemiological studies noted the contributions of vestibular pathologies to unhealthy aging (29–31), as well as the impact of age on the recovery of vestibulo-ocular and balance control functions after unilateral vestibular failure in humans (32). Behavioral analysis of vestibular-mediated performance in young and older mice has been assessed previously (33). In the present study, we used a recently developed mouse model of vestibular disorder to compare the abilities of balance and posture restoration between young adult and old mice following unilateral vestibular damage. Specific signs of alterations in vestibular function, such as circling, head tilt, and muscle dystonia, were monitored at different time points over 3 weeks following the TTK administration. General behavior was also evaluated over the same period by assessing the quality of both exploration and walking, body height and the number of rearings. We present here, in a well-controlled animal model of unilateral vestibular disorder, novel data showing that young adult and elderly mice are together able to restore normal postural-locomotor function following transient unilateral vestibular excitotoxic insult, but do so at different time courses.

## Materials and methods

### Animals

We used 23 C57Bl6/N mice originating from our own breeding of parent mice from Charles River laboratories (L'arbresle, France). Elderly mice (*n* = 11) were 22 months of age when injured using either the TTK procedure (*n* = 5) or sham treated (*n* = 6). Young mice (*n* = 12) were 3 months of age when injured using either the TTK procedure (*n* = 6) or sham treated (*n* = 6). All animals were kept in standard animal cages under conventional laboratory conditions (12 h/12 h light-dark cycle, temperature: 22 ± 2°C, humidity: 55 ± 5%) with *ad libitum* access to food and water. The behavior experiments were conducted during the light phase. The experimental protocols and animal care were in compliance with the institutional guidelines (council directive 87/848, October 19, 1987, Ministry of Agriculture and Forestry, Veterinary Department of Health and Animal Protection) and international laws (directive 2010/63/UE609, 13 February 2013, European Community) and policies (personal authorization #I-67UnivLouisPasteur-F1-04 for RC). The present study was approved by the Neuroscience Ethics Committee N°71 from the French National Committee on animal experimentation.

### Surgical procedure

Once the mice were deeply anesthetized using isoflurane gas, we created a hole in the eardrum using a micro needle (30 gauge) and infused 20 μL of 25 mM kainate (Abcam ab120100, dissolved in NaCl 0.9% and with a pH level adjusted to 7.4). Once the trans-tympanic instillation was done, we kept the mouse on its side for 30 min (the lesion side was on the top). We used the same procedure for the sham mice but 0.9% NaCl was substituted for kainate.

### Behavioral exploration

**Figure 1:**
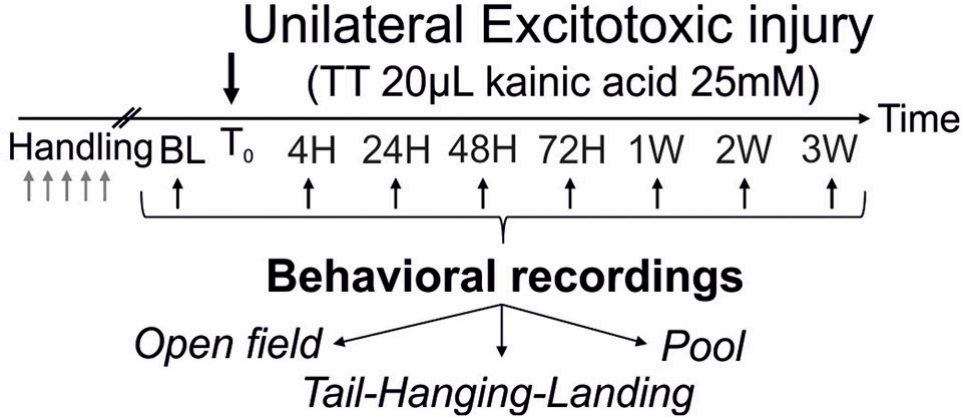
Operational protocol. After the trans-tympanic administration of kainate (TTK) performed in young or old mice (3 or 22 months of age respectively), assessment of the behavioral consequences of the vestibular insult was performed at 7 time points after the lesion (4 h, 24 h, 2 days, 3 days, 1 week, 2 weeks, 3 weeks) using 3 different paradigms (open-field, tail-hanging landing and pool).

A 5-day handling period was implemented to habituate the animals to manipulation (2 min per day for each mouse). The behavioral evaluations were performed at 8 times points: baseline (BL), 4 h (4 h), 24 h (24 h), 48 h (48 h), 72 h (72 h), 1 week (1 W), 2 weeks (2 W) and 3 weeks (3 W) as shown in Figure 1. Except for the number of rearings and for the swim duration, the other items were quantified on a scale from 0 (mouse exhibited no trouble at all) to 3 (value that represents the highest degree of vestibular alteration), as previously described (26–28, 34) and based on a previous paper that showed the utility of the behaviors evaluated (35). All measurements were performed systemically by the same experimenter both directly and offline through analyze of the videos recorded for each experiment. For each item considered, the scoring method was previously validated by three different persons in order to check the reproducibility between experimenters. Videos were used to perform a second quantification, in order to confirm or infirm previous observations.

### Devices and behavioral parameters

Behavioral assessment of vestibular function was performed using an open field (OF) and a pool. The OF consisted of a Plexiglas square with 30-cm sides fixed on a bottomless table. We used a Plexiglas box (length: 34 cm, width: 21 cm and height: 20 cm) filled with water as a pool, which was maintained at 32°C to avoid hypothermia.

In the OF, spontaneous animal behaviors were recorded over 2 min and quantified two sets of parameters: (1) items linked to specific signs of vestibular function alterations and (2) behaviors relative to the global state of the mice (referred to as general behavior). Signs of vestibular alterations included *circling* (stereotyped movement in circles around the hips of the animal), *head tilt* (inclination of the head), *muscle dystonia* (hypertonia on the side of the lesion), *tumbling* (mice turned on themselves around their central axis; scored from none to uninterrupted spins) and *head bobbing* (abnormal intermittent backward extension of the neck; scored from none to compulsive movement). General behavior encompassed the following items: *quality of horizontal* (if the mouse crossed the device – score 0- or not at all - score 3), *vertical exploration* (if the mouse straightened up frequently or not), *quality of walking* (from assured -0-, to null - score 3), and *body height* (the mouse tended to lower the height of their body in cases of dizziness). The *number of rearings* was not included in the global vestibular score, as we performed simple addition of the redresses number. The *tail-hanging-landing* test consisted of holding the animal by the tail and lifting it vertically over a height of ~50 cm. This test reactivated vestibular syndrome as a result of both macular and canalar systems stimulation (especially as the animals were spinning when lifted) and simultaneous withdrawal of proprioceptive and plantar information. The quality of the forelimb extension normally produced to reach the ground was also monitored (i.e. quality of the landing that was scored from -0- perfect preparation of the two front paws, to -3- absence of preparation) as well as the *axial body rotation* (twisted body item from no rotation -0- to continuous twisting -3-). This paradigm induced a syndrome reactivation after mouse landed. Intensity of this syndrome reactivation was quantified as the accentuation or the reappearance of vestibular signs (circling, head tilt, muscle dystonia, bobbing) from -0- (no sign) to -3- (max expression/accentuation of vestibular signs).

To assess their ability to maintain their balance in water and to monitor the quality of their swimming, the mice were put in the pool for 30 s. Evaluation of the swim quality was performed using the specific behavior grid detailed previously (28). We also paid attention to whether the mice were tumbling or not in the water. Once the mice were removed from the water, we monitored the intensity of syndrome reactivation (i.e. an accentuation or a recovery of vestibular signs, as previously detailed in the THL part) and their grooming skills (ability to clean and dry itself). The effective swim duration was recorded. This referred to the time spent swimming before we took the mouse out of the water. Detailed descriptions of all these behavioral parameters were provided previously (28).

### Statistics

SPSS (IBM Corp. Released 2016. IBM SPSS Statistics for Windows, Version 24.0. Armonk, NY: IBM Corp.) was used for all statistical analyses. Performances recorded across the different paradigms were analyzed using a two-between, 1-within factor ANOVA, which considered the factor of “time” (time points 1–8 i.e., BL, 4 h, 24 h, 48 h, 72 h, 1 W, 2 W, and 3 W) as the within factor and “treatment” (V, vehicle vs. TTK) and “age” (young vs. old) as the between factors. The ANOVA was followed by Bonferroni multiple-comparisons test when appropriate. For all the ANOVAs reported, violations of the sphericity assumption (homogeneity of covariance) were corrected using the Greenhouse-Geisser procedure; the corrected *P*-value along with the epsilon correction factor (ε) are reported. As the ε was lower than 0.75 in all analyses, we used G-G corrected *P*-values for all the data presented. To analyse kainate effect (“treatment”) and the age effect (“age”), Student's *t*-test was performed for independent groups (sham *vs*. treated mice and young *vs*. elderly treated-mice). To facilitate the reading of the analyses, we focused only on the age effect on the treated mice using Bonferroni's multiple-comparisons test (elderly-TTK mice *vs*. young-TTK mice). The results are expressed as the means ± SEM. Values with *p* < 0.05 were considered significant. Lack of significant effect was indicated by the letters *ns*.

## Results

Acute vestibular syndrome (AVS) was induced in young adult and elderly mice via trans-tympanic administration of kainate (TTK) according to the method detailed previously (28). A schematic representation of the experimental design is shown in Figure 1. Values are given in mean score + SEM.

### Comparative effect of TTK administration on vestibular function in young adult and elderly mice

**Figure 2:**
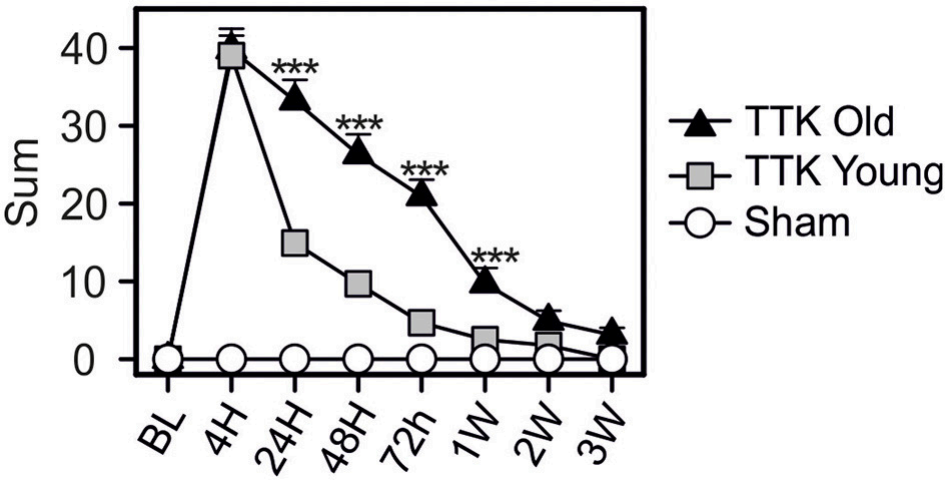
Temporal variation of the vestibular score sum. Sum of all items obtained in the open-field during spontaneous exploration (circling, head tilt, muscle dystonia, bobbing, tumbling, vertical/horizontal explorations, walk quality), in the Tail Hanging Landing paradigm (quality of the landing, axial rotation of the body and the intensity of the reactivation) and in the pool (swim quality, tumbling intensity, syndrome reactivation intensity and finally grooming skills). Injured-elderly mice are represented by dark triangles, injured-young mice by gray squares and the sham-group by open circles. Results are expressed as means + SEM. ANOVA repeated measures followed by Bonferroni multiple-comparisons test were used to observe AGE effect in injured mice (i.e TTK elderly vs. TTK young): ***p < 0.001 (*n* = 5 TTK-elderly, *n* = 6 TTK-young and *n* = 12 sham mice).

The global vestibular function of the lesioned and control mice was assessed in both young and elderly mice by monitoring several behavioral parameters that are detailed in the Material and Methods section. Figure 2 illustrates the variations over time in the sum of all the recorded parameters. Both groups of young and old, lesioned mice displayed altered vestibular function within the first 4 h after the TTK administration. The amplitude of the altered vestibular behavior did not significantly differ between the two groups at 4 h (39.8 ± 2.7 in old mice; 39 ± 2.6 in young mice). However, its change over time significantly differed depending on age [*F*_(1, 19)_ = 31.2, *p* < 0.001]. Elderly mice displayed higher vestibular scores than the young mice did at 24 h, 48 h, 72 h, and 1 W (*p* < 0.001, at all the time points).

### Effect of TTK administration on specific signs of vestibular alterations in young and elderly mice

**Figure 3:**
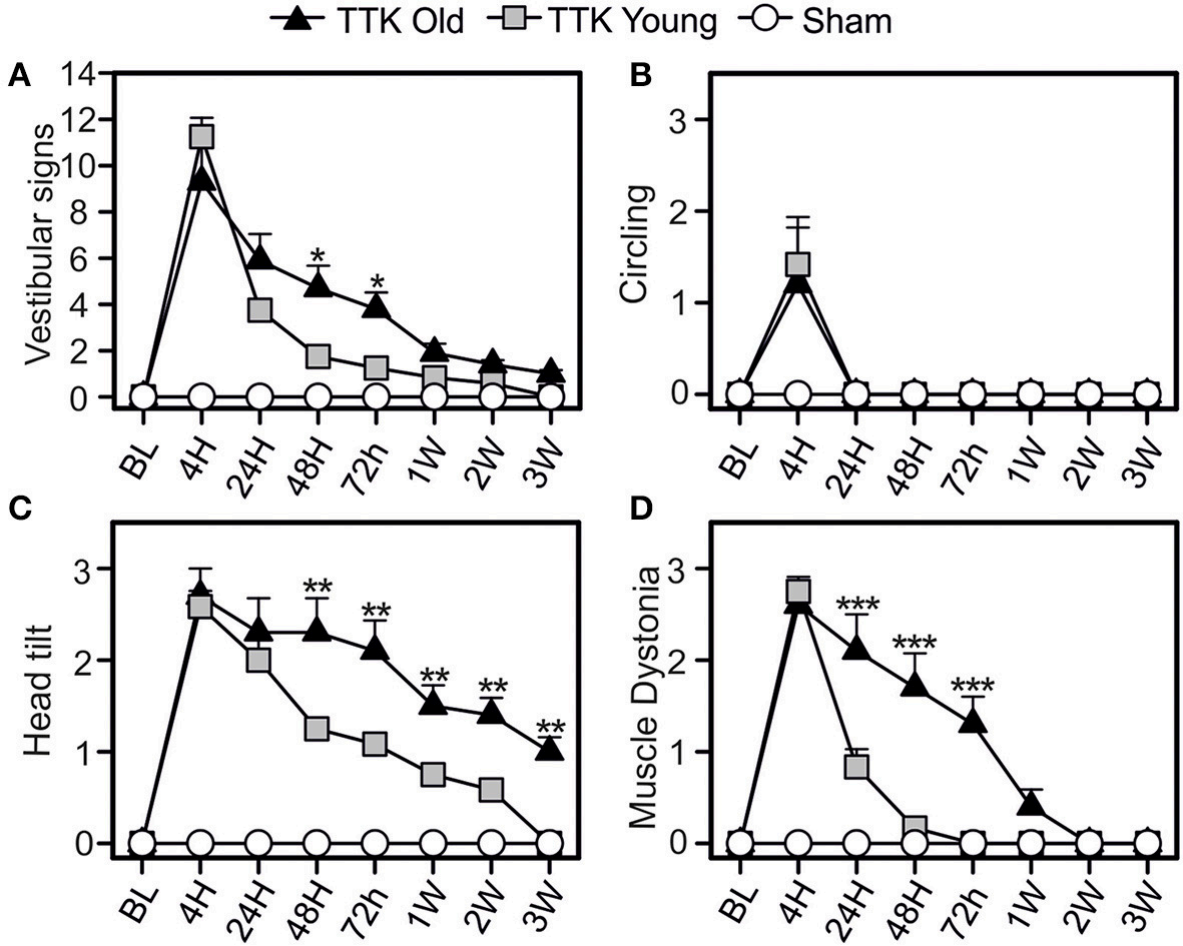
Change of vestibular specific signs in the open field over time. Measurements of the global expression of vestibular specific signs were done **(A)** thanks to three specific items: the circling **(B)**, the head tilt **(C)**, and the muscle dystonia **(D)**. Results are expressed as means + SEM. The injured-elderly mice are represented by dark triangles, the injured-young mice by gray squares and the sham-group by open circles. ANOVA repeated measures followed by Bonferroni multiple-comparisons test to observe AGE effect in injured mice (i.e. TTK elderly vs. TTK Young): *p < 0.05; **p < 0.01; ***p < 0.001 (*n* = 5 TTK-old, *n* = 6 TTK-young and *n* = 12 sham mice).

We first analyzed specific signs of vestibular alterations (sum of the scores obtained for circling, head tilt, muscle dystonia, bobbing and tumbling items) using the open field (Figure 3). We observed vestibular deficits in all the treated animals, which peaked at 4 h (11.3 ± 0.8 and 9.3 ± 1.8 for the young and elderly mice, respectively) and was followed by a sharp decline at 24 h (3.7 ± 0.3 and 5.9 ± 1.1 for the young and elderly mice, respectively) until reaching a baseline level at 3W in the young mice (Figure 3A). Significant deficit could be recorded 3 W after the lesion in the old mice (1 ± 0.1, *p* < 0.001 compared to the baseline level). Statistical effects of age [*F*_(1, 19)_ = 4.50, *p* < 0.05; the Bonferroni's multiple-comparisons test showed significant differences at 24 and 48 h, *p* < 0.05] and kainate infusion [*F*_(1, 19)_ = 135.5, *p* < 0.001] were observed on the vestibular sign alterations. To better characterize the impact of age on vestibular lesions, we analyzed each of the items used to calculate the sum. Circling behavior was first examined (Figure 3B). The mean score peaked at 4 h (1.4 ± 0.4 and 1.2 ± 0.7 for the young and elderly mice, respectively) and then directly returned to the baseline level at 24 h in both treated groups. We also observed a treatment effect [*F*_(1, 19)_ = 8.1, *p* < 0.01], showing that the kainate administration induced a significant disturbance without an age effect [*F*_(1, 19)_ = 0.05, *ns*], indicating that this item was similarly perturbed in the young–and elderly treated mice. We then examined the change of head tilt (Figure 3C). The mean score peaked at 4 h in both treated groups (2.6 ± 0.2 and 2.7 ± 0.4 for the young and elderly mice, respectively). No significant differences were observed at this specific time point; however, over time, the variations in head tilting significantly differed between the young and the elderly mice [*F*_(1, 19)_ = 11.89, *p* < 0.01]. While the young treated mice displayed significant head tilts until 3 W, the elderly presented significantly higher scores at all times points (*p* < 0.01). The time course change of lesion-induced muscle dystonia was also monitored over the different time points (Figure 3D). The two groups of treated mice displayed similar peaks in the alteration at 4 h (2.7 ± 0.2 and 2.6 ± 0.2 in the young and elderly treated mice, respectively) but again, with a different evolution over time [*F*_(1, 19)_ = 16.3, *p* < 0.001]. Significant differences between the young and elderly mice at 24, 48, and 72 h (*p* < 0.001) were measured. All together, these observations indicated that aging significantly altered the restoration of specific vestibular functions, such as head tilt and muscle dystonia.

### Impact of TTK administration on the general behavior of young and elderly mice

**Figure 4:**
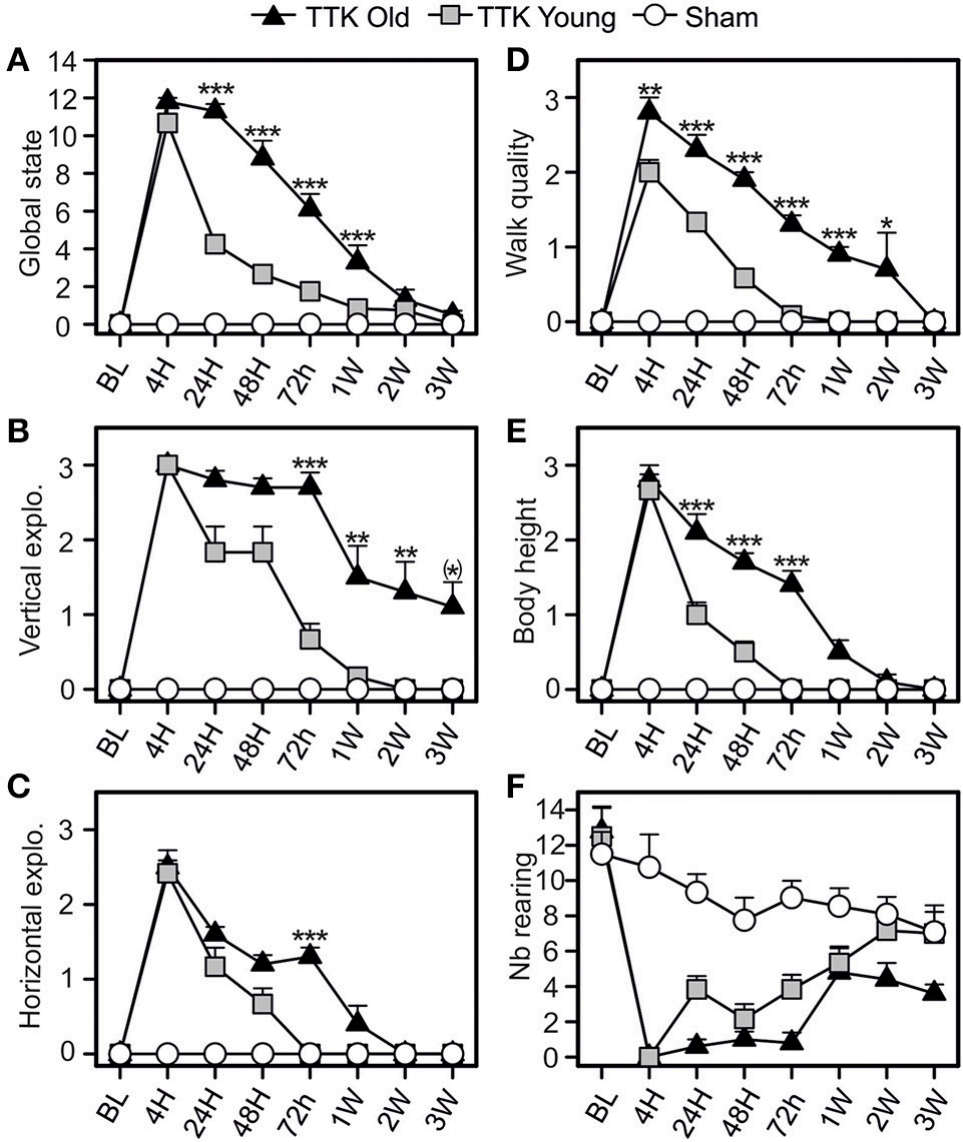
Temporal evolution of the global state of the mice. The global state **(A)** of the mice was evaluated by combined several items: the quality of vertical **(B)** and horizontal exploration **(C)**, the walk **(D)**, the body height **(E)**, and the number of redress **(F)**. Results are expressed as means + SEM. The injured-elderly mice are represented by dark triangle, the injured-young mice by gray square and the sham-group by open circle. ANOVA repeated measures followed by Bonferroni multiple-comparisons test to observe AGE effect in injured mice (i.e. TTK elderly vs. TTK Young): *p = 0.06; *p < 0.05; **p < 0.01; ***p < 0.001 (*n* = 5 TTK-elderly, *n* = 6 TTK-young and *n* = 12 sham mice).

The consequences of the unilateral vestibular insult on the general behavior of the young and elderly mice were evaluated by monitoring the horizontal and vertical explorations, walking quality and body height (Figure 4). Alterations in general behavior peaked 4 h following the TTK administration (10.7 ± 0.8 and 11.8 ± 0.2 in the young and elderly mice, respectively; Figure 4A), no significant difference between the two groups was observed at this specific time point. Conversely, the decline in this value over time significantly differed between the young and elderly mice [*F*_(1, 19)_ = 45.1, *p* < 0.001]. A significant difference was observed between the young–and elderly treated mice up to 1 W post-injury, *p* < 0.001 for all the time points. The mean score of the vertical exploration (Figure 4B) peaked at 4 h for each of the two groups of treated mice without significant difference being observed (3 ± 0 in the young and elderly mice). Its decline over time was significantly slower in the old mice [*F*_(1, 19)_ = 19.6, *p* < 0.001] and specifically differed at 72 h, 1 W, 2 W, and 3 W (*p* < 0.01 and *p* = 0.06 at 3 W). Considering the quality of horizontal exploration (Figure 4C), peaks in the alterations for both ages were observed at 4 h (young mice, 2.4 ± 0.2 and elderly mice, 2.5 ± 0.2) and were followed by a significant decrease in deficits that varied depending on age [*F*_(1, 19)_ = 8.8, *p* < 0.01]. Elderly injured mice differed from young mice at 72 h (*p* < 0.001). Walking quality was also highly altered in both groups (Figure 4D). Statistically significant differences (*p* < 0.01) between the elderly (2.8 ± 0.2) and young (2 ± 0.2) injured mice were noticed until 2 W. Young injured mice improved walking quality scores faster than old injured mice [*F*_(1, 19)_ = 62.9, *p* < 0.001], with specific differences depending on age being observed at all the time points except at 3 W (*p* < 0.05 or less). We observed a transient impact of the vestibular insult on body height (Figure 4E), with a peak in the alteration at 4 h without any significant difference between the young and elderly injured mice (2.6 ± 0.2 and 2.8 ± 0.2, respectively). However, the decline over time in this value significantly differed between the two groups [*F*_(1, 19)_ = 40.4, *p* < 0.001] with significant differences at 24, 48, and 72 h (*p* < 0.001). The number of rearing's (Figure 4F) was altered in both groups [*F*_(1, 19)_ = 16.3, *p* < 0.001], but we did not observe an age effect [*F*_(1, 19)_ = 0.1]. However, statistical interaction between the two variables of age and time [*F*_(7, 133)_ = 2.4, *p* < 0.05] was observed, meaning that the change over time in rearing was different depending on the age of the treated mice. The elderly treated mice recovered less than the young treated mice, and this was more marked at the last time point, i.e., 3 W (7.1 ± 1.5 in sham mice; ± 1.2 in young treated mice; 3.6 ± 0.5 in old-treated mice). All these results revealed a peak in the alterations at 4 h in both of the treated groups, followed by a recovery of all parameters that was delayed in the elderly mice.

### Comparative effects of the TTK administration on the swimming capacities of the young and elderly mice

**Figure 5:**
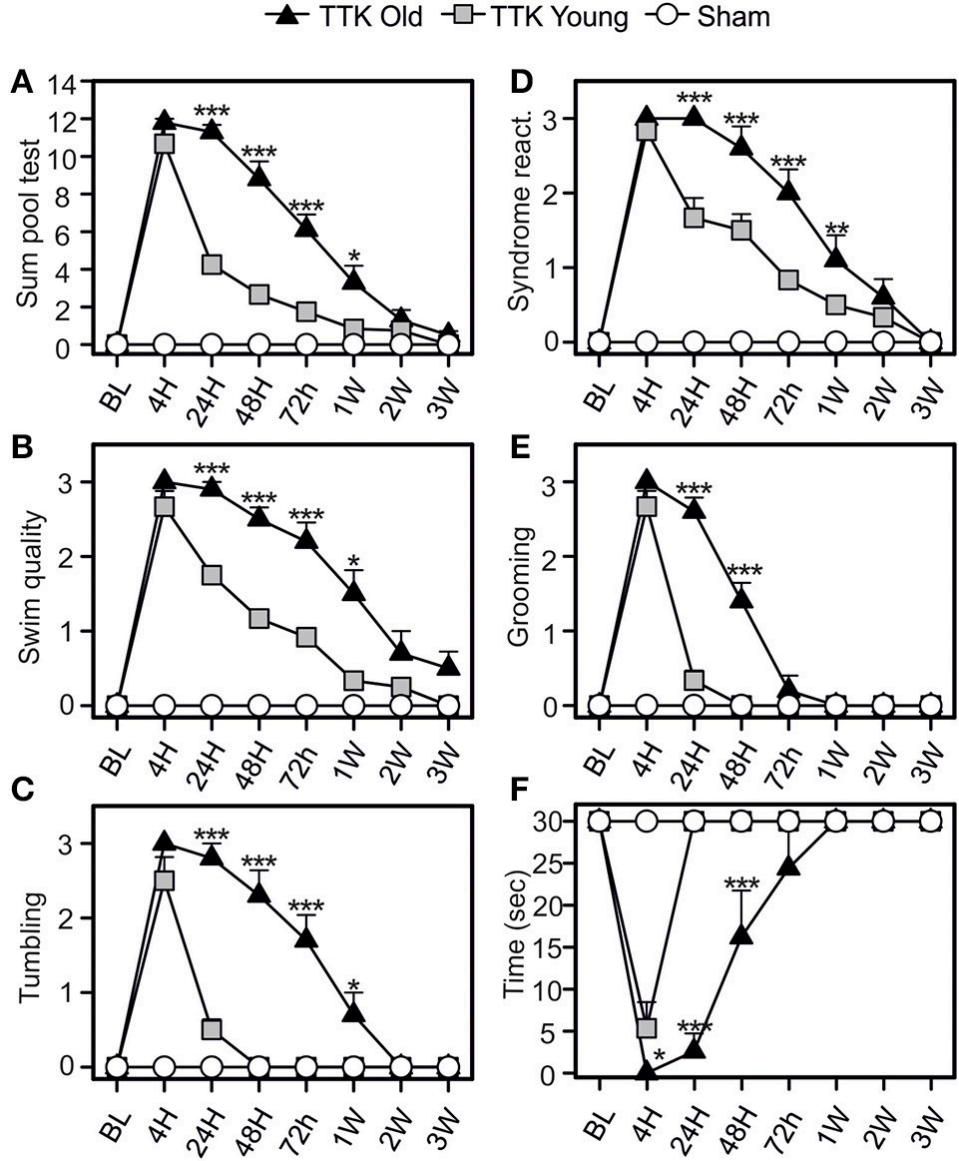
Change of vestibular score depending on the time in the pool test. The swimming abilities of the mice in the pool test were first calculated in a global way (**A** sum of four items) and then detailed through four different items **(B–E)**: the swim quality **(B)**, if mice were tumbling or not **(C)**, the intensity of syndrome reactivation **(D)**, and the grooming skills **(E)** of mice once removed from the water. Swim duration was also assessed **(F)**. Results are expressed as means + SEM. The injured-elderly mice are represented by dark triangles, the injured-young mice by gray squares and the sham-group by open circles. ANOVA repeated measures followed by Bonferroni multiple-comparisons test to observe AGE effect in injured mice (i.e. TTK elderly vs. TTK Young): *p < 0.05; **p < 0.01; ***p < 0.001 (*n* = 5 TTK-elderly, *n* = 6 TTK-young and *n* = 12 sham mice).

The pool test was used to analyse the effect of partial withdrawal of proprioceptive inputs in the water (Figure 5). We calculated a global score, which was obtained by summing the independent scores of four items: the quality of the swim, the expression of tumbling behavior and, once the mice were removed from the water, the intensity of syndrome reactivation and their grooming skills. We also monitored the swim duration. The global score (Figure 5A) peaked at 4 h (10.6 ± 0.8 and 11.8 ± 0.2 in the young and elderly treated mice, respectively; *ns*) and was followed by a gradual decline over time that differed between the two groups of treated mice [*F*_(1, 19)_ = 37.6, *p* < 0.001]. Significant difference between young and elderly injured mice at 24 h and 1 W after the injury (*p* < 0.05 or less) was observed. The quality of the swim (Figure 5B) was altered for both groups of treated mice after the TTK administration and peaked at 4 h (2.6 ± 0.2 and 3 ± 0 in the young and elderly treated mice, respectively). The recovery capacities were influenced by the age of the mice [*F*_(1, 19)_ = 24.4, *p* < 0.001], as the young mice recovered faster than the elderly mice did. We observed significant differences at all time points between 24 h and 1 W (*p* < 0.05 or less). The score related to the measures of tumbling in the water peaked at 4 h (2.5 ± 0.3 in the young and 3 ± 0 in the elderly treated mice, Figure 5C). The large decrease differed depending on age [*F*_(1, 19)_ = 48.3, *p* < 0.001]. Significant impact of age on the treated mice at all time points between 24 h and 1 W (*p* < 0.05 or less) was observed.

Syndrome reactivation (Figure 5D) reached its peak at 4 h (2.8 ± 0.1 for the young mice and 3 ± 0 for the elderly mice). We found significant differences between the young and elderly injured mice [*F*_(1, 19)_ = 15.3, *p* < 0.001] and, more specifically, differences at 24, 48, and 72 h (*p* < 0.001). The grooming skills of the mice (Figure 5E) were altered in both groups, with a peak at 4 h (2.6 ± 0.2 and 3 ± 0 for the young and elderly mice, respectively) followed by different recoveries depending on age [*F*_(1, 19)_ = 51.6, *p* < 0.001]. These differences were marked during the short post-lesion delays (24 and 48 h, *p* < 0.001). The swim duration (Figure 5F) was highly altered at 4 h for both groups (5.3 ± 3.1 for the young mice while elderly mice unable to swim at all). The young injured mice recovered faster than the elderly mice did [*F*_(1, 19)_ = 34.2, *p* < 0.001], with significant differences being observed at all the time points until 48 h (*p* < 0.001). Altogether, these data confirmed an impact of the unilateral vestibular insult on the swimming ability of the mice, which extended when they were removed from the water (specifically, their grooming skills) and that was higher in the elderly than in the young injured mice.

### Higher sensitivity to the tail-hanging-landing (THL) test in elderly mice after unilateral vestibular damage

**Figure 6:**
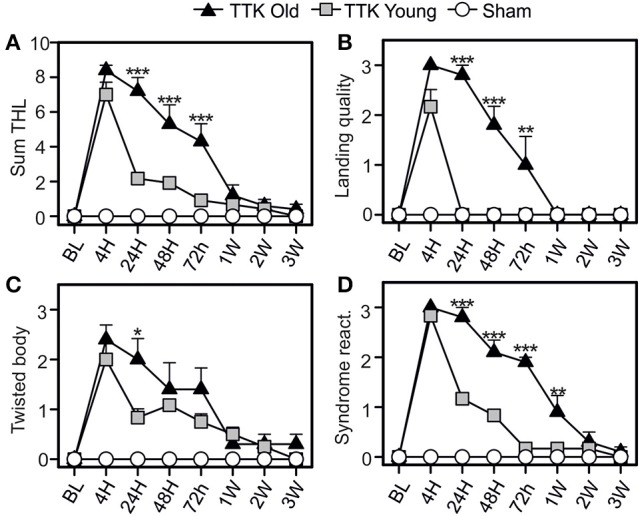
Temporal variation of vestibular score in the tail-hanging-landing paradigm. A global score was calculated **(A)** through the combination of three items **(B–D)**: the quality of the landing **(B)**, the axial rotation of the body (**C** degree of twisted body) and the intensity of syndrome reactivation **(D)** once landing. Results are expressed as means + SEM. The injured-elderly mice are represented by dark triangles, the injured-young mice by gray squares and the sham-group by open circles. ANOVA repeated measures followed by Bonferroni multiple-comparisons test to observe AGE effect in injured mice (i.e. TTK elderly vs. TTK Young): *p < 0.05; **p < 0.01; ***p < 0.001 (*n* = 5 TTK-elderly, *n* = 6 TTK-young and *n* = 12 sham mice).

The impact of the TTK administration on elderly and young mice was compared using the specific tail-hanging-landing (THL) paradigm (Figure 6). We first calculated a global score (Figure 6A) and then continued with the analysis by distinguishing the three items. The quality of landing (Figure 6B), position of each mouse's body when they were moving (Figure 6C) and intensity of the syndrome reactivation after landing (Figure 6D) were assessed. The mean scores that were obtained with the THL (Figure 6A) revealed that both treated groups exhibited an important alteration at 4 h (12.4 ± 5.5 and 12.4 ± 5.5 in young and elderly mice respectively), which was followed by a different decrease depending on the age of the mice [*F*_(1, 19)_ = 13.5, *p* < 0.01]. Significant differences between the two groups of treated mice was observed at each of the time points from 24 to 72 h (*p* < 0.001). The quality of the landing highlighted a clear impact of the treatment, with a peak in the alterations being observed at 4 h in both groups (mean scores: young treated, 2.1 ± 0.3 and elderly treated mice, 3 ± 0). We found a global impact of age [*F*_(1, 19)_ = 37.9, *p* < 0.001], and significant differences between the young and elderly treated mice at 24, 48, and 72 h (*p* < 0.01). The peak in the alterations for the “twister body” parameter (Figure 6C) was obtained at 4 h for both groups (mean scores: 2 ± 0.3 and 2.4 ± 0.3 in the young and elderly treated mice, respectively). We did not observe a significant global effect of age [*F*_(1, 19)_ = 1.9, *ns*]. The syndrome reactivation score was higher at 4 h (2.8 ± 0.1 and 3 ± 0, in young and elderly treated mice, respectively). Global impact of age [*F*_(1, 19)_ = 27.2, *p* < 0.001] was noticed. While we observed only a tendency at 4 h (*p* = 0.07), the impact of age was clearly measurable from 24 h to 1 W post-injury (*p* < 0.001).

To summarize, except for the twirl reaction of the body, significant difference was consistently observed between the scores of the young treated mice and the ones expressed by elderly injured mice.

## Discussion

This study was designed to analyse how acute vestibular syndrome (AVS) is expressed in elderly mammals and how its characteristics differ from those of young adults. The originality of our exploration relies on the fact that we used a paradigm in which transient excitotoxic-based unilateral vestibular insults were performed.

No specific etiology is currently commonly accepted to explain the generation of the AVS. However, its sequence and symptoms, especially those expressed in the static phase, suggest a unilateral and sudden loss of the sensory input from peripheral vestibular sensors. Different animal models have been developed in the past to reproduce the sequence and characteristics of the AVS. Among these, the use of selective blockers of voltage-gated Na^+^ channels such as lidocaine (36, 37) or TTX (38, 39), by temporarily blocking peripheral sensory input, allowed to reproduce most of the vestibulo-oculomotor or post-locomotor alterations encountered in the AVS. The area of contact between the inner ear sensory cells and the fibers forming the 8th cranial nerve is now pointed out as the most vulnerable area of the ear. At the level of the cochlea, the nerve segment between the auditory primary neurons and the inner hair cells is the most specifically affected upon aging (40, 41). Selective excitotoxic damage of primary cochlear synapses also appears as the key element in cases of sound over stimulation (25), as well as in case of local ischemia (42). Intracochlear damages may also be involved in a majority of sensory neural hearing loss (24). Several authors believe that an intralabyrinthine process is massively underestimated or ignored in the peripheral vestibulopathy (PVP) (43, 44). It can therefore be assumed that selective damage to the vestibular primary synapses could support all of the symptoms experienced in PVP.

With the aim to reproduce these excitotoxic damages, we developed in rodents a method of transtympanic administration of glutamate receptors agonists (26). By combining electron microscopy and immunohistochemical approaches, we confirmed that the local excitotoxic environment generated by the strong afflux of glutamate-receptors agonist evoked selective destruction of the synaptic contacts between vestibular hair cells and primary neurons forming the vestibular nerve (26, 27). Swellings and retractions of the postsynaptic terminals were interpreted as the consequence of the massive influx of cations and water through the AMPA-kainate receptors. These histological damages were accompanied by functional manifestations characteristic of the AVS, such as spontaneous nystagmus and posture-locomotor deficits (26, 27). Subsequently, the inner ear damages progressively resolved within a week after the insult initiation. This repair of the primary synapses was concomitant with a reduction of the vestibular symptoms (26). These observations pointed out the TTK model as a suitable model to follow the AVS induced by damages to peripheral synapses, conversely to previous models in which the process of functional restoration rather relied on the relieve of the block by the pharmacological agents.

Age correspondence between mice and human is now widely recognized, especially regarding neural development. Mice of 3 months are considered as equivalent at human of 20–26 years, while mice of 22 months are considered as equivalent of human of about 70 years (45).

TTK administration evoked posturo-locomotor alterations with similar characteristics in the young adult and elderly mice. In both cases, this condition is composed of two main phases, which are differentiated both by their kinetics and by the amplitude of the expressed symptoms. Vestibular-specific signs and alterations of general behavior are present in both populations. During the acute phase, which began immediately after the TTK administration and peaked within the first hour after the vestibular impairment, the various symptoms are expressed all together with their respective intensities at their paroxysm. At this time, no difference was observed in terms of amplitude of the evoked symptoms between young adult and elderly mice. This suggested that the induction process of the unilateral vestibular damage, together with its impact on postural-locomotor behavior are age-independent. Subsequently, the different symptoms gradually regressed, each according to its own sequence. In the young adult mice, typical symptoms, such as circling or muscular dystonia, regressed quickly and had almost disappeared in few days following the vestibular insult. Other signs, such as head tilt (which corresponds to a typical clinical sign encountered in vestibular patients), take between 1 and 2 weeks to disappear. These postural-locomotor alterations also affect different activities of general behavior, such as walking, vertical and horizontal exploratory behaviors and body position. Most of these behavioral changes regress within the first few h after the insult, while others, such as vertical exploration, take longer to recover, as was previously reported (28). In the elderly mice, except for the circling behavior, the decline of both the vestibular-specific signs and the alterations of the general behavior significantly differed relative to young adult mice. In the case of vestibular-specific signs, full withdrawal of muscle dystonia operates, with a delay of several days, while head tilt was still maintained 3 W after the vestibular insult. Most of the alterations in general behavior also fully disappeared, but with a delay of several days compared to young adult mice. Similarly, restoration of normal swimming ability, reduction in associated altered behaviors, and disappearance of symptoms induced by the THL test occurred, but with longer delay than that of young adult mice.

In the young adult rat subjected to TTK administration, we previously demonstrated (using thorough electron microscopy observation of damaged synapses) that spontaneous reafferentation of vestibular sensors predominantly takes place by the end of the first week after the excitotoxic insult (27). 24h after the insult, most synapses still displayed features of non “fully-repaired” synapses (i.e. calyx terminals not fully reorganized). However, significant reduction of the AVS was obvious at this time. We proposed that the early compensation phase of static vestibular signs was independent on the synaptic repair process and it rather depends on central processes probably beneficiating from the inputs of spared vestibular synapses. We also suggested that the subsequent and full reduction of the vestibular syndrome was mainly supported by the vestibule reafferentation. In present study we observe similar sequence of vestibular syndrome reduction in the young adult mouse. Interestingly, the effectiveness of the early compensation was dramatically reduced in the aged mice (i.e.: 2/3 of vestibular static signs compensated at 24 h in young mice *vs*. only 1/3 in aged mice–Figure 3A). This may tentatively explain by the loss/slowing down of the cellular/molecular mechanisms involved the early compensation. However, full reduction of most of the recorded vestibular specific and general behavior signs (excluding head tilt and walk quality) was observed in the elderly mice as soon as 1W after the insult initiation. This suggests that the spontaneous repair of vestibular primary synapses is probably maintained in aged mice. Whether the effectiveness of this last process is reduced will need further histological investigations.

Neuronal plasticity and synapse preservation both decline with aging in particular neuronal subpopulations. This has been illustrated both in central [(46) for review] and peripheral nervous systems (47). Swelling and loss of synaptic contacts, decreases in postsynaptic density sizes, and altered mitochondrial dynamics are features typically found in the aged brain (48). In the inner ear, age-related decline in both the density of hair cells and in vestibular primary neurons has been reported in humans, as in rodents (49–56). Deciphering the respective contributions of peripheral synaptic repair and central compensation in the restoration of postural-locomotor function has now become the next major challenge. Finally, another non-exclusive hypothesis is the reduction in the physical properties (muscular mass, body flexibility, and the ability to move) in the elderly mouse that may prevent proper restoration of posture and balance.

Interestingly, a similar difference in the restorative capacities of the VOR and the equilibrium was observed between very young (18 months to 9 years old) and young (9–15 years old) children that presented acute unilateral vestibular deafferentation (uVD) diagnosed as vestibular neuritis–observations in 47 children (Majer, submitted). vertigo, vomiting and postural symptoms resolved before any recovery of the VOR (and faster in the youngest group) suggesting that compensatory mechanisms develop more efficiently for static parameters than for dynamic parameters. VOR to rotatory chair were the first dynamic parameters to recover much before the caloric test and the vHIT suggesting that functional recovery, as well as compensatory processes, develops better in the most frequently used velocity ranges of head movement (middle velocity head rotations on rotatory chairs). These results showed that 93% of the children recovered vestibular function at 1 year, while in the literature, <50% of the adults had recovered 1 year after uVD (57) and suprisingly 50% of the adults and children displayed functional recovery. This could suggest similar processes for recovery in adults and children with a limiting critical period of plasticity in adults and elderly. Spontaneous restoration of vestibular function assessed by vHIT is often observed after vestibular neuritis (58). However, neither the cellular support of this endogenous process, nor its age-dependency have been elucidated so far. In children, recovery and compensation are fast (<1 week) while in adults the mean range of recovery and/or compensation occurs on the order of weeks. This suggests that peripheral restorations of vestibular function, somatosensory/visual substitution and central compensation occur more rapidly in children than adults.

## Conclusion

The present study, carried out using a well-controlled animal model of vestibular lesions, highlighted a significant effect of age on the restoration of the postural-locomotor and balance functions after a unilateral excitotoxic-type vestibular insult. The results raised important questions regarding the respective contributions of peripheral synaptic repair and central compensation to the restoration of postural-locomotor function following unilateral vestibular deafferentation. This animal study opens new avenues for future investigations on how the early postural and locomotor disturbances following a unilateral insult are compensated for by various plasticity mechanisms, and in which extend age influences these mechanisms.

## Author contributions

RC, BT, and CC: conceptualization; RC and AE: data curation; CC: funding acquisition; RC, AE, BT, and CC: investigation; CC: supervision; RC, SW-V, BT, AE, and CC: writing - review and editing.

### Conflict of interest statement

The authors declare that the research was conducted in the absence of any commercial or financial relationships that could be construed as a potential conflict of interest.
